# Multiscale Aspects of Generation of High-Gamma Activity during Seizures in Human Neocortex^1^,^2^,^3^

**DOI:** 10.1523/ENEURO.0141-15.2016

**Published:** 2016-05-23

**Authors:** Tahra L. Eissa, Andrew K. Tryba, Charles J. Marcuccilli, Faiza Ben-Mabrouk, Elliot H. Smith, Sean M. Lew, Robert R. Goodman, Guy M. McKhann, David M. Frim, Lorenzo L. Pesce, Michael H. Kohrman, Ronald G. Emerson, Catherine A. Schevon, Wim van Drongelen

**Affiliations:** 1 Committee on Neurobiology, The University of Chicago, Chicago, Illinois 60637; 2 Departments of Pediatrics, The University of Chicago, Chicago, Illinois 60637; 3 Department Physiology, Medical College of Wisconsin, Milwaukee, Wisconsin 53226; 4 Department of Neurological Surgery, Columbia University, New York, New York 10032; 5 Department Neurosurgery, Medical College of Wisconsin, Milwaukee, Wisconsin 53226; 6 Department of Neurosurgery, Mount Sinai Hospital, New York, New York 10029; 7 Department Surgery, The University of Chicago, Chicago, Illinois 60637; 8 The Computation Institute, The University of Chicago, Chicago, Illinois 60637; 9 Department of Neurology, Columbia University, New York, New York 10032; 10 Committee on Computational Neuroscience, The University of Chicago, Chicago, Illinois 60637

**Keywords:** epilepsy, HFOs, human, modeling, neocortex, seizure

## Abstract

High-gamma (HG; 80-150 Hz) activity in macroscopic clinical records is considered a marker for critical brain regions involved in seizure initiation; it is correlated with pathological multiunit firing during neocortical seizures in the seizure core, an area identified by correlated multiunit spiking and low frequency seizure activity. However, the effects of the spatiotemporal dynamics of seizure on HG power generation are not well understood. Here, we studied HG generation and propagation, using a three-step, multiscale signal analysis and modeling approach. First, we analyzed concurrent neuronal and microscopic network HG activity in neocortical slices from seven intractable epilepsy patients. We found HG activity in these networks, especially when neurons displayed paroxysmal depolarization shifts and network activity was highly synchronized. Second, we examined HG activity acquired with microelectrode arrays recorded during human seizures (*n* = 8). We confirmed the presence of synchronized HG power across microelectrode records and the macroscale, both specifically associated with the core region of the seizure. Third, we used volume conduction-based modeling to relate HG activity and network synchrony at different network scales. We showed that local HG oscillations require high levels of synchrony to cross scales, and that this requirement is met at the microscopic scale, but not within macroscopic networks. Instead, we present evidence that HG power at the macroscale may result from harmonics of ongoing seizure activity. Ictal HG power marks the seizure core, but the generating mechanism can differ across spatial scales.

## Abstract (precis)

High-gamma (HG; 80-150 Hz) activity in macroscopic clinical records is considered a marker for critical brain regions involved in seizure initiation; it is correlated with pathological multiunit firing during neocortical seizures in the seizure core, an area identified by correlated multiunit spiking and low frequency seizure activity.

## Visual Abstract

**Figure:**
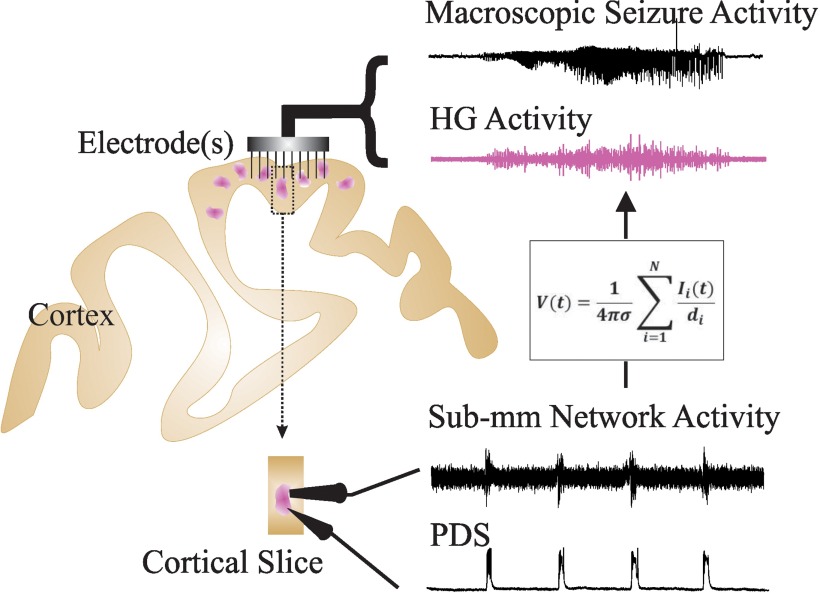

## Significance Statement

We demonstrate that ictal HG power (80-150 Hz) in cortical measurements is increased in the seizure core and appears on clinical recordings as a result of volume conduction and synchrony between harmonics generated during ongoing seizure activity. The accuracy of ictal HG activity to localize the core is superior to that of the lower-frequency seizure activity since these are generated across much larger cortical areas. Therefore, the detection of HG power provides a promising tool for the surgical evaluation of patients with epilepsy.

## Introduction

Epilepsy is a common neurological disorder, affecting ∼1% of the population of the world. Importantly, one third of the patients in whom epilepsy is diagnosed will not respond to medication (Kwan and Brodie, 2000). For drug-resistant epilepsy, the potential treatment options are limited to vagus nerve/deep-brain stimulation or surgical removal of seizure-initiating tissue. Focal epilepsy is an extremely heterogeneous disease (Berg et al., 2010). Therefore, identifying the seizure onset zone (SOZ) can be a nontrivial undertaking (Rosenow and Lüders, 2001). Current practice uses low-frequency local field potentials (LFPs) from electrocorticographic (ECoG) recordings to define the SOZ, but these slower rhythms can occur across a wide area of cortex and may overestimate the SOZ. Several studies have suggested that activity in the beta (16-30 Hz) and gamma ranges (30-80 Hz) as well as high-frequency activity between 80 and 500 Hz, both during seizures (ictal) and between seizures (interictal), can help to localize the SOZ (de Curtis and Gnatkovsky, 2009; Modur et al., 2011; Fujiwara et al., 2012; Jacobs et al., 2012; Schevon et al., 2009; Zakaria et al., 2012; Einevoll et al., 2013; Weiss et al., 2013; Cho et al., 2014). A subrange of high-frequency activity from 80 to 150 Hz, termed high-gamma (HG) activity here, is of particular interest given that activity in this frequency range has been related to epileptic activity (Miller, 2010; Einevoll et al., 2013), and specifically correlated with pathological multiunit burst firing during neocortical ictal activity (Schevon et al., 2012; Weiss et al., 2013). While pathological HG activity is a promising tool for detection of the SOZ, the effects of the spatiotemporal dynamics of seizure on HG power generation are not well understood.

Physiological HG activity has been well described and appears to relate to the synchronization of activity from inhibitory interneurons, gap junctions, and excitatory synapses onto interneurons (Buzsáki, 2006; Bartos et al., 2007; Ray and Maunsell, 2010, 2011; Buzsáki et al., 2012). In contrast, various hypotheses exist for the development of ictal HG activity, and very little is known about the precise relationships among HG activity, pathological synchrony, and seizure generation (Menendez de la Prida and Trevelyan, 2011). While some hypotheses about ictal HG generation suggest mechanisms similar to those that describe physiological activity (Traub et al., 2001; Trevelyan 2009), ictal HG activity has also been correlated with pathological small clusters of pyramidal cells and the presence of paroxysmal depolarizing shifts (PDSs) in populations of neurons (Bragin et al., 2000; Grenier et al., 2003).

PDSs are considered a hallmark of seizure activity at cellular scale. A neuron is considered to be in the PDS state when the soma membrane is strongly depolarized, ∼30 mV above resting potential. At this level of depolarization, neuronal action potentials have a significant decrease in intraburst spike rate and spike amplitude (Li, 1959; Matsumoto and Marsan, 1964a,b; Marcuccilli et al., 2010). Recently, a theoretical study provided compelling evidence that PDSs, which previously had been confirmed only in experimental models of epilepsy, are also present during human ictal activity (Meijer et al., 2015). Additionally, this study suggested that PDSs were localized to an area of brain tissue termed the core, characterized by high levels of correlation between multiunit spike activity and LFP amplitude. While PDSs are markers of cellular epileptic activity and HG activity is representative of epileptic network activity, the unique relationship linking the two scales in human seizures has yet to be determined.

Here, we test the hypothesis that ictal HG activity in the seizure core is related to pathological PDS activity generated in small (submillimeter) networks. Furthermore, we propose that ictal HG activity at the clinical macroelectrode (centimeter scale) is due to a combination of volume conduction and synchronized low-frequency seizure activity. To accomplish a multiscale analysis, we investigate the PDS–HG activity relationship at the microscale in an *in vitro* model of human epileptic cortex. Next, we use *in vivo* microelectrode array (MEA) recordings of human seizure activity to link HG power in microelectrode and macroelectrode signals. Results are evaluated using a modeling approach to mathematically describe the relationships among neuronal activity, locally synchronized network activity, and macroscopic HG activity. We present and discuss evidence that PDS activity is associated with increased HG activity, and confirm that pathological, increased synchrony plays a role in the development of HG power. However, since synchrony levels decrease with network size, we suggest different mechanisms underlying HG power in microelectrode versus macroelectrode recordings.

## Materials and Methods

### Neocortical brain slice preparations and electrophysiological recordings

Seven surgery patients (four female, three male; Table 1) in this study met the standard of care criteria for pharmacologically intractable epilepsy, which was defined as having persistent epileptic seizures that do not cease after treatment with three or more antiepileptic drugs. The area targeted for resection was identified as the seizure focus using subdural electrocorticography. In all cases, it took <10 min to transport the surgically removed neocortical tissue from the operating room to the research laboratory. The tissue was transported in ice-cold artificial CSF (ACSF) saturated with medical grade carbogen (95% O_2_ plus 5% CO_2_). ACSF contained the following (in mm): 118 NaCl, 3 KCl, 1.5 CaCl_2_, 1 MgCl_2_ * 6H_2_O, 25 NaHCO_3_, 1 NaH_2_PO_4_, and 30 D-glucose, equilibrated with carbogen (95% O_2_ plus 5% CO_2_, pH = 7.4). All chemicals used to prepare the ACSF were obtained from Sigma-Aldrich.

**Table 1.** Clinical and demographic data for slice tissue

**Patient number**	**Slice number**	**Activity type**	**Age at time of resection (years)/sex**	**Tissue sample location**	**Pathology**	**Seizure type**
1	S1	PDS	12/F	Left temporal	Cortical dysplasia	Complex partial, generalized tonic-clonic
	S2	PDS		Parietal- occipital		
2	S3	PDS	8/M	Left occipital	Cortical dysplasia	Complex partial
	S4	Burst				
3	S5	PDS	5/F	Right frontal	Nonspecific	Complex partial
4	S6	Burst	8/F	Right anterior frontal	Nonspecific	Complex partial Left clonic
5	S7	Burst	17/F	Left parietal	Cortical dysplasia	Complex partial, simple partial
6	S8	Burst	15/M	Left temporal	Cortical Dysplasia	Complex partial
7	S9	Burst	18/M	Right frontal	Mild neuronal ischemia	Complex partial
	S10	PDS				
	S11	PDS				
	S12	PDS				

Burst, Neurons show nonsaturating burst firing; F, female; M, male.

Neocortical slices (400 µm thick) were prepared with a VT1000S Microtome (Leica) and were stored in carbogen-saturated ACSF at room temperature (22˚C). Slices were used within 10 h following resection. During recordings, human neocortical brain slices were submerged under circulating ACSF, saturated with carbogen in recording chambers (flow rate, 15 ml/min; total circulating volume, 200 ml; recording chamber volume, 6 ml). Experiments were performed at 30 ± 0.7°C using a TC-344B Temperature Regulator with an in-line solution heater (Warner Instruments). We raised ACSF [K+] from 3 to 5 mm to enhance rhythmic activity Tryba et al. 2011; Marcuccilli et al. 2010; van Drongelen et al. 2006.

The internal review board committees at the Medical College of Wisconsin approved all *in vitro* experiments. Informed consent was obtained from all patients, and surgeries and treatment plans were not directed by or altered as a result of these studies.

### *In vitro* recording and experimental seizures from human brain slices

Whole-cell current-clamp recordings of regular spiking human neurons were obtained with a Multi-Clamp 700B Amplifier (Molecular Devices), filtered at <10 kHz, and digitized at 20,000 samples/s. During patch-clamp recordings, the discharge pattern of each neuron was first identified in cell-attached mode and remained similar in whole-cell configuration. Once in whole-cell configuration, neurons were evaluated on a number of criteria and accepted for use only if they had a baseline membrane potential below −58 mV, access resistance <30 MΩ, and input resistance >50 MΩ. The baseline membrane potential was corrected for the liquid junction potential calculated using pClamp 10 Software (Molecular Devices). Patch-clamp electrodes were manufactured from filamented borosilicate glass tubes (Clark G150F-4, Warner Instruments). Current-clamp electrodes were filled with an intracellular solution containing the following (in mm): 140 K-gluconate, 1 CaCl_2_ * 6H_2_O, 10 EGTA, 2 MgCl_2_ * 6H_2_O, 4 Na_2_ATP, and 10 HEPES with a resistance between 3 and 5 MΩ. To induce rhythmic population bursts and/or seizure-like activity (SLA), GABA receptors were blocked by bath application of 10 µm bicuculline-free base, and 15 µm NMDA was additionally bath applied (Sigma-Aldrich; Marcuccilli et al. 2010). Bath application of these drugs resulted in seizure-like bursting in seven brain slices and nonepileptiform activity in five cases.

**Figure 1.**
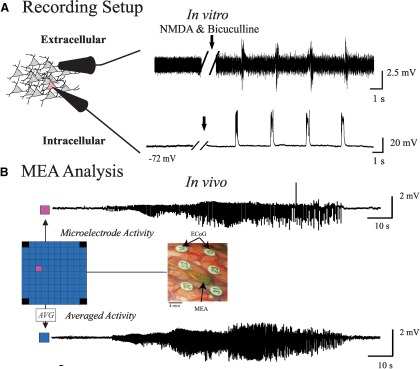
Methodology. ***A***, Acute slice recording setup with concurrent extracellular and intracellular recordings. SLA and PDSs were induced with the addition of bicuculline and NMDA (black arrow). ***B***, Local seizure activity from patients was recorded with MEAs placed concurrently with cortical grids that measured the ECoG. The power of the HG activity was determined in both individual microelectrode signals (magenta) and averaged MEA activity (blue).

Simultaneous extracellular population recordings to monitor network population activity within human neocortical slices were performed (Fig. 1A). Extracellular multiunit population (network) recordings were obtained with glass suction electrodes (filled with ACSF) positioned on the brain-slice surface within ∼50 µm of the whole-cell current-clamp recordings (Fig. 1A). The extracellular signals were amplified 10,000-fold and filtered between 3 Hz and 1 kHz using a preamplifier built in-house and a Model P-55 AC Amplifier (Grass Technologies/Astro-Med). The data were digitized at 20 kHz with a Digidata Acquisition System (Molecular Devices) and stored on an IBM-compatible PC using Molecular Devices Software (Med, Inc.).

### *In vivo* recordings

Study participants Columbia University consisted of four patients (two female, two male) with pharmacoresistant focal epilepsy who underwent long-term intracranial EEG studies to help identify the epileptogenic zone for subsequent surgical removal. All *in vivo* experiments were approved by the internal review board committees at Columbia University. Prior to obtaining and using data for this study, informed consent was obtained. The patients’ surgeries and treatment plans were not directed by or altered as a result of these studies.

A 96-channel, 4 × 4 mm microelectrode array (Utah array, Blackrock Microsystems) was implanted along with subdural electrodes with the goal of recording from SOZs. Signals from the microelectrode array were acquired continuously at 30 kHz per channel (0.3 Hz to 7.5 kHz bandpass; 16-bit precision; range, ±8 mV). The reference was epidural. ECoG signals were acquired using a standard clinical video EEG system (Xltek, Natus Medical) with a bandwidth of 0.5-500 Hz (Fig. 1B).

The HG data shown in Figure 8C were filtered off-line using a second-order Butterworth band filter of 80-150 Hz. Up to three seizures in each patient were selected for detailed analysis, to avoid biasing the dataset from the patients in whom many seizures were recorded (Table 2). Channels and time periods including artifacts were excluded.

**Table 2.** clinical and demographic data for patients implanted with MEAs

	**P1 (32 years/F)**	**P2 (19 years/F)**	**P3 (30 years/M)**	**P4 (39 years/M)**
Implant location	Left lateral and subtemporal	Right lateral and subtemporal, parietal, occipital	Left lateral frontal, mesial frontal, temporal	Left lateral and mesial frontal
MEA location	Left inferior temporal gyrus 2.5 cm from anterior temporal pole	Right posterior temporal, 1 cm inferior to angular gyrus	Left supplementary motor area, 3 cm superior to Broca’s area	Left lateral frontal 2 cm superior to Broca's area
Seizure onset zone	Left basal/anterior temporal, (including MEA site)	Right posterior lateral temporal, (including MEA site)	Left supplementary motor area (including MEA site)	Left frontal operculum (3 × 3 cm cortical area, including MEA site)
Days recorded	5	28	4	4
Number of seizures analyzed	3	1	1	3
Seizure Types	Complex partial	Complex partial with secondary generalization	Complex partial/tonic	Complex partial
Pathology	mild CA1 neuronal loss; lateral temporal nonspecific	Nonspecific	N/A (multiple subpial transections performed)	Nonspecific

F, Female; M, male; N/A, not applicable; P, patient.

**Table 3.** Statistics table

	**Data structure**	**Type of test**	**95% confidence intervals**
A–Extracellular SLA vs baseline	Bootstrapped	Difference of the means	32e3–137e3 µV^2^
B–Intracellular PDS vs baseline	Bootstrapped	Difference of the means	0.82e7–2.1e7 µV^2^
C–PDS vs normal bursting	Bootstrapped	Difference of the means	−2.6e7 to 1.4e7 µV^2^
D–SLA vs non-SLA	Bootstrapped	Difference of the means	21e3–116e3 µV^2^
E–Extracellular synch vs asynch	Nonparametric	Wilcoxon signed-rank	1–1*
F–Intracellular synch vs asynch	Nonparametric	Wilcoxon signed-rank	1–1*
G–Extracellular asynch vs baseline	Nonparametric	Wilcoxon signed-rank	0.25–1
H–Intracellular asynch vs baseline	Nonparametric	Wilcoxon signed-rank	0.25–1
I–Core vs penumbra microelectrodes	Bootstrapped	Difference of the means	0.49e5–4.2e5 µV^2^
J–Core vs penumbra averaged activity	Nonparametric	Wilcoxon rank sum	1–1*
K–Core vs penumbra percentage difference	Nonparametric	Wilcoxon rank-sum	1–1*

*Distributions are perfectly separated.

### Analysis

#### Detection of epileptiform activity

We used intracellular recordings from *in vitro* data to categorize records as SLA if bursts exhibiting PDSs were consistently observed (*n* = 7), and as control if the recordings lacked PDSs (*n* = 5). A burst showing a PDS is characterized by a decrease in the amplitude of the individual action potentials and saturation plateaus (Li, 1959; Matsumoto and Marsan, 1964a,b; Marcuccilli et al., 2010). Each recording included simultaneously recorded network activity. Amplitude thresholds were used to detect bursts using custom-made scripts in MATLAB (MathWorks). Intracellular bursts were detected by applying a two-point threshold of ≥40 mV at *t* = 0 and ≤50 mV at *t* = −100 ms (100 ms prior). Extracellular bursts were detected using a threshold of >2 mV. All detections were verified by reviewing the signals. *In vivo* seizure recordings were categorized as “core,” an area defined by correlated low-frequency and multiunit activity during the seizure, or as “penumbra,” which includes surrounding tissue that is unrecruited. Thus, we distinguished core and penumbra using the procedure reported by Weiss et al. (2013).

#### Volume conduction and signal averaging

Volume conduction effects of brain activity are described by a quasistatic solution of Maxwell’s equations, meaning delays attributed to volume conduction may be ignored. Therefore, the compound effect of multiple generators, such as HG activity at the macroscale, can be modeled as a weighted linear combination of the local currents as follows (Nunez and Srinivasan, 2006):

(1) $$V(t)=\frac{1}{4\pi \sigma }\sum_{i=1}^{N}\frac{I_{i}(t)}{d_{i}},$$

where *I_i_*(*t*) is the current of the *i*th source at time *t*, *d_i_* is the distance between the electrode and the *i*th current source, and σ is the tissue conductivity. Because of the linearity of the volume conduction process, the frequencies of the locally generated oscillations are preserved when their combined effect is recorded by an electrode [i.e. only amplitude and phase (and not frequency) can be affected in linear combinations; van Drongelen, 2007]. Obviously, the magnitude of the combined effect of generators at a particular frequency critically depends on the phase distribution of the signals (i.e. their level of synchrony). Because the weights in the average will not affect our assessment of the modeled synchrony effects, we have simplified Equation 1 by using a straightforward unweighted average (i.e., by using uniform weights).

Although *in vitro* bursting patterns were variable, we consistently recorded either PDS or non-PDS bursting. In our analysis, we used the PDS as a marker for SLA and non-PDS activity as control. To evaluate the compound effects of multiple local sources, we used the serially recorded bursts in the slices. For the *in vivo* data, this step was not required since the multiple locations were truly recorded in parallel. Using the above approach to implement the formalism in Equation 1, 200 ms epochs from the *in vitro* recordings were extracted and averaged to create four types of datasets. Set 1 consisted of a single average of synchronous activity, using the bursts as the synchronizing trigger (see Fig. 4A,*B*, black traces). Set 2 was a single average of desynchronized activity created by randomized selection of epochs from recordings (see Fig. 4A,*B*, green traces). Set 3 was a single signal representing baseline activity, averaged from the randomized selection of epochs in recordings acquired prior to bursting. Set 4 consisted of 500 averaged signals. These were obtained by controlled application of jitter to the individual epochs (trials) in the average (for details, see the Jitter implementation section).

For the *in vivo* data, we produced only two of the sets described above. As depicted in Figure 1B, set 1 was obtained by creating an averaged of the microelectrodes, and the equivalent to set 4 consisted of 200 jittered averages. 

#### Jitter implementation

**Figure 2.**
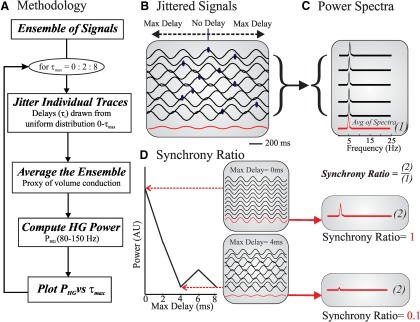
Jitter analysis and synchrony. Cartoon of the jitter analysis in which a randomized delay (shift) was applied to each signal prior to computing the signal average. The jitter simulates levels of asynchrony between the signals, while the average represents the effect of volume conduction (Eq. 1). ***A***, Flowchart of jitter methodology. Each signal within the ensemble is jittered by applying a randomized delay (τ_i_) ranging from 0 to τ_max_. The jittered signals are then averaged to represent the compound activity of the signals due to volume conduction. We perform power spectral analysis on the averaged activity and determine the amount of HG power in the signal. The amount of HG power is then plotted against the maximum delay (τ_max_; i.e., the degree of asynchrony). This procedure is repeated for a range of increasing maximum delays (in this example, a range from 0 to 8 ms in steps of 2 ms). ***B***, Set of randomly jittered signals (blue dots mark the center of each signal) drawn from a uniform distribution characterized by a maximum delay, τ_max_. The red trace is the time average of the jittered dataset. ***C***, The power spectra of the individual signals in the inset of ***A***, and the average of these power spectra (in red). ***D***, The relationship between the power in a time-averaged signal and the degree of asynchrony determined by the maximum delay (τ_max_) of the applied jitter. The two gray panels depict example datasets: one in which the signals are perfectly synchronized (No jitter, i.e., Max Delay = 0) and one with significant jitter (maximum delay = 4 ms). To quantify the amount of synchrony in a set of signals, we computed the synchrony ratio by dividing the power from the time-averaged power spectrum by the power from the averaged power spectra (i.e., the values marked as 2 in ***D*** divided by those marked by 1 in ***C***).

For our jitter analysis, we produced a set of averages in which random delays were applied to epochs of recorded data prior to averaging and then analyzed for HG content (Fig. 2A). An example can be seen in Figure 2B. The shifted epochs (black traces) are averaged (red trace) to represent compound activity with a level of synchrony governed by the maximal amount of jitter (Max Delay). The Max Delay determines the maximum of the uniform distribution for drawing the random delays. We created 500 averages for the *in vitro* dataset, with a different maximum delay ranging from 0 to 1000 ms in 2 ms steps. Since we used the average as a proxy for volume conduction (Eq. 1), the maximum delay represented the degree of synchrony or desynchronization in the network (Fig. 2D). For the *in vivo* dataset, we used a similar analysis to create a set of 200 averages with maximum delays that ranged from 0 to 200 ms (in 1 ms steps).

To further examine the results of the jitter analysis, we developed a set of three alternative models in which we determined how multiple signals in an ensemble generate power within the HG band of their compound signal. In Model I, we determine the effect of a set of sinusoidal signals within the HG band, Model II is used to determine the effects of pairs of sinusoidal signals, and Model III describes the effect of an ensemble of nonsinusoidal signals. To be able to determine the effect of asynchrony across the ensemble on the HG power, we then applied a controlled level of jitter to the individual signals using a procedure identical to the one applied to the experimental traces described above (Fig. 2). Thus, both the experimental data and the model used jitter across an ensemble of signals to mimic asynchrony between generators that contribute to a compound signal (where the contribution of the individual generator to the compound signal is governed by volume conduction). For further details on the model development, see Results.

#### Power spectra and synchrony ratio

To reduce the possibility of overestimating HG power, power spectra of individual and averaged signals were estimated using the multitaper power spectral density analysis, which minimizes noise and spectral leakage across frequencies (Thomson, 1982). To account for differences in amplitude between seizure activity in core and penumbra in the *in vivo* dataset, these recordings were normalized using the sum of squares of the seizure epoch prior to computing the power spectra. Total HG power was obtained by summing the power in the 80-150 Hz band.

To assess synchrony levels across the MEA electrodes, HG power was determined using the following two methods: (1) the HG band power from the average of the spectra of the individual microelectrodes (Fig. 2C, red trace); and (2) the power from the spectrum of the averaged time domain signals (Fig. 2D, two example red traces). Since the average from the power spectra does not include phase information, while time averages do, the power obtained in method 2 divided by the one obtained in method 1 reflects the level of synchrony. For example, if the synchrony between locally generated signals is perfect (i.e., the phases of all trials in the average are identical), the power spectrum of the averaged signal is equal to the averaged power spectrum of the individual signals (Fig. 2, Max Delay = 0 ms), as follows:

(2) $$P\left[\frac{1}{N}\sum_{i=1}^{N}x_{i}(t)\right]=\frac{1}{N}\sum_{i=1}^{N}P\left[x_{i}(t)\right],$$

where *x_i_*(*t*) is one of the *N* locally generated oscillations at location *i*, and *P*[…] denotes taking the power spectrum of the expression in between the square brackets. However, if synchrony is less than perfect, the relationship in Equation 2 changes due to phase differences (i.e., jitter) between the individual signals (Fig. 2, Max Delay = 4 ms), thus:

(3) $$P\left[\frac{1}{N}\sum_{i=1}^{N}x_{i}(t)\right]<\frac{1}{N}\sum_{i=1}^{N}P\left[x_{i}(t)\right],$$

and the ratio between the expression left and right of the < sign can be used to quantify synchrony on a scale of 0 to 1, as follows:

(4) $$S=\frac{P\left[\frac{1}{N}\sum_{i=1}^{N}x_{i}(t)\right]}{\frac{1}{N}\sum_{i=1}^{N}P\left[x_{i}(t)\right]}.$$

To avoid spurious results, we considered only this synchrony ratio if the HG power of the time domain average during the seizure activity exceeded that of the preictal activity by 5 Standard Deviations.

### Statistics

Data obtained from the same patient as well as from activities in the same slice preparation may include dependencies because they originate from the same sources. To correct for this possible dependency, and thus to avoid underestimating HG variability from *in vitro* bursts/baseline epochs and the *in vivo* microelectrode, signals were analyzed using hierarchical bootstrapping. The technical term for this standard statistical procedure is “resampling with replacement.” Briefly, to estimate the mean and SEM, we generated 5000 datasets by randomly selecting data points from our original datasets (e.g., the amount of HG power detected in each burst from a single slice recording or in each microelectrode recording within a given patient). Since our data show a two-tier hierarchical structure, with the first tier comprising a specific slice per patient and the second tier comprising the HG power detected, we first sampled with replacement for the first tier and then sampled again with replacement for the second. The means of these 5000 sets were then used as an estimate of the sampling distribution of the sample mean. Then, looking at the distribution of the means from the resampled datasets, we could obtain an estimate of the mean and SEM for our recorded data. A full description of this standard nonparametric method is beyond the scope of this article; therefore, we refer to Efron and Tibshirani (1994) and Gallas et al. (2009) for further details.

In order to determine whether the means between the different recorded data were statistically significantly different (e.g., intracellular PDS activity vs intracellular baseline), we took another 5000 resampled sets using instead the demeaned data, in essence forcing the bootstrapped data to satisfy the null hypothesis of being identical, and generated a null hypothesis distribution. From these null hypothesis distributions, we could then determine a *p* value based on the difference of the means between two groups. These *p* values can now be estimated as the following proportion:

(10) $$\frac{\begin{array}{c} \#\;\mathrm{resampled mean differences from null in modulus}\;\geq \;\mathrm{observed mean difference} \end{array}\;}{\mathrm{total number of resampled sets}}$$

The numerator represents the number of times the null hypothesis failed to be true (i.e., the difference between the means from the null distribution is greater than the original observed mean difference). The denominator represents the total number of bootstrapped sets that were tested. Significance was determined if <5% of the samples failed the test.

To test whether HG power was different between the synchronous and asynchronous datasets, we used the nonparametric Wilcoxon signed-rank test, which accounts for the paired nature of the data. Unpaired data, such as averaged MEA recordings from the core and penumbra, were compared using a rank sum test (Kvam and Vidakovic, 2007).

## Results

### *In vitro* data

#### Neurons generate HG activity during seizures

**Figure 3.**
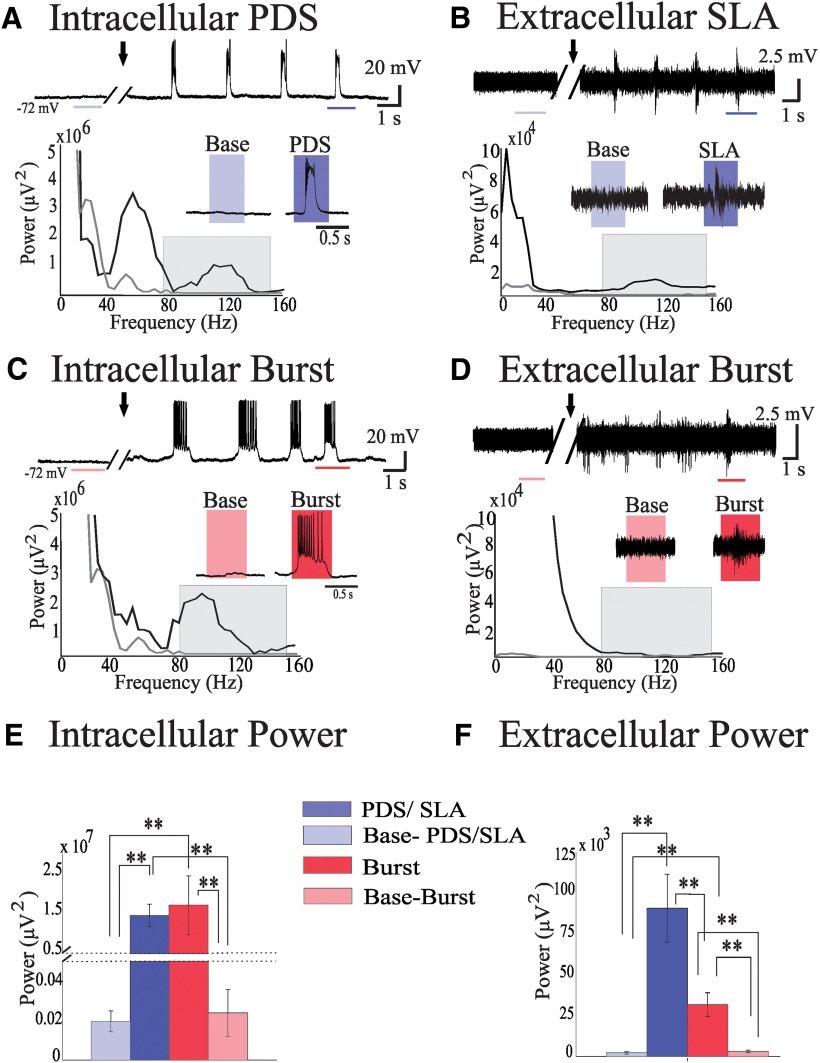
Seizure-like activity produces significant amounts of HG power. ***A***, Example trace of intracellular activities recorded in an acute slice of human neocortical tissue. The arrow marks bath application of bicuculline and NMDA. Insets show examples of PDS and baseline (Base) signals. Intracellular PDSs are characterized by large depolarizations of ∼30 mV above the resting potential that includes depolarization block. This resulted in reduced interburst spike rates, compared with physiological bursts, and decreased spike amplitudes during the burst. Power spectra of intracellular signals show increased power in the HG band (80-150 Hz, gray box) during PDS activity (black trace) compared with baseline (gray trace). ***B***, Same as in ***A*** for the extracellular signal recorded simultaneously with the intracellular signal. Insets depict SLA and network baseline activity (Base). Power spectra show an increase in the HG band power during SLA compared with baseline. ***C***, Intracellular, nonsaturating bursting activity from acute human neocortical slice. Baseline (Base) and bursting (Burst) activities are shown in the insets. Power spectra show increased HG power during bursting over baseline. ***D***, Extracellular bursting activity simultaneously recorded in the same slice as ***C***. Power spectra do not show a significant increase of HG power during the network burst. ***E***, Bar graph of intracellular HG power across states, including the mean HG power and SEM, shows a large increase in HG power during both PDSs and nonsaturating bursts. Note the discontinuity in the vertical axis. Dark blue, PDS; light blue, corresponding baseline activity; red, nonsaturating bursts; pink, corresponding baseline activity. ***F***, Bar graph of extracellular HG power across states shows that extracellular recordings from networks with SLA (that included single neurons with PDSs) show significantly more HG power than network recordings from nonsaturating cellular bursts. Dark blue, SLA; light blue, corresponding baseline activity; red, network bursting of networks with nonsaturating cellular bursts; pink, corresponding baseline activity. **p < 0.02.

To study the HG-generating capability of small networks, we recorded experimentally induced SLA (*n* = 7) that included intracellular PDSs and simultaneous extracellular network activity in human neocortical brain slices (Fig. 3A,*B*, top traces). From these recordings, pathological activity was detected, extracted, and compared to baseline activity recorded before experimental onset of SLA (Fig. 3A,*B*, PDS, SLA, and baseline insets). Power spectra indicated that the experimental seizure activity resulted in a huge increase in HG power (80-150 Hz) compared with baseline, both in the intracellular and extracellular signals (Fig. 3A,*B*,*E*,*F*; *p* < 0.05^A,B^).

#### HG power is increased in small networks during PDS activity compared with nonsaturating bursting networks

We compared the power of HG activity seen during pathological SLA with the amount produced by slices under the same experimental conditions that did not show SLA (*n* = 5). Intracellular recordings from neurons in brain slices that did not generate SLA exhibited normal, nonsaturating, bursts rather than PDSs (Fig. 3C). Single neurons displaying PDSs produced amounts of HG power similar to those of neurons generating normal (non-PDS) bursting (Fig. 3E; *p* = 0.63^C^); however, extracellular recordings of the local networks generating SLA compared with non-SLA networks produced significantly more HG power (*p* < 0.05^D^; Fig. 3B,*D*,*F*).

#### Synchrony can sustain HG power in a larger network

**Figure 4.**
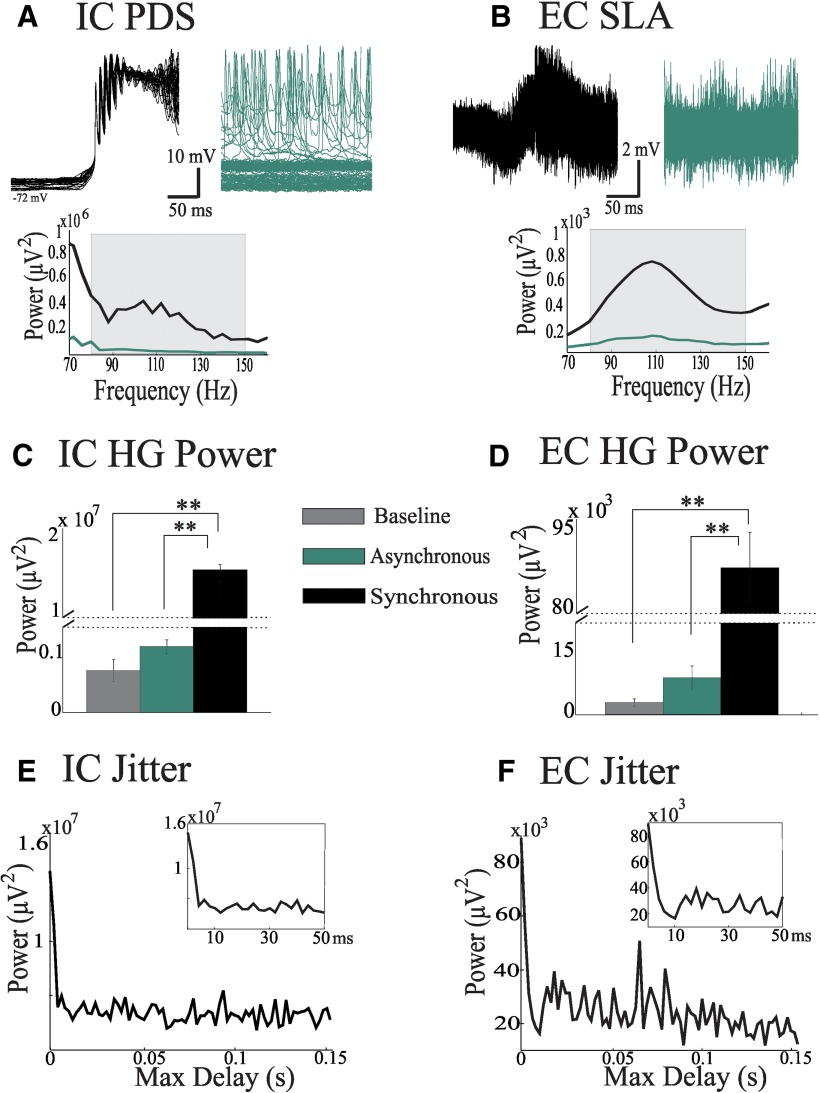
Power of average HG activity depends on synchrony levels. ***A***, ***B***, Sequential burst events from intracellular (IC) PDS activity (***A***) and extracellular (EC) SLA slice recordings (***B***) were detected and used to represent signals generated across multiple locations. Detected burst events (synchronous case, black traces) or randomly selected epochs (asynchronous case, green traces) were then superimposed to mimic volume conduction. These superimposed signals were then averaged, and power spectra were computed (bottom plots; black, synchronous case; green, asynchronous case; gray boxes, HG band 80-150 Hz). ***C***, ***D***, Bar graphs of the mean HG power and SEM of the averaged signals (gray, baseline; green, asynchronous average; black, synchronous average). Result shows a large difference in the amount of HG power (note the discontinuity in the vertical axes) between the synchronous and asynchronous scenarios (**p < 0.02). ***E***, ***F***, Jitter plots showing the amount of HG power retained in a series of averages in which the superimposed signals undergo randomized delays prior to averaging. For each average, these randomized delays are drawn from a uniform distribution ranging from zero to Max Delay (abscissa). Insets detail the drop in HG power retained with maximum delays of <50 ms. Both IC and EC recordings show a dramatic drop in HG power with maximum delays of <10 ms.

We then tested whether HG activity in a macroscopic cortical area might be explained by volume conduction of synchronized local HG activity from multiple sources by averaging burst-triggered signals from our *in vitro* data (Fig. 4A,*B*, black traces). We also averaged randomly extracted epochs to produce a set of data mimicking volume conduction during asynchronous neural activity (Fig. 4A,*B*, green traces). Both the intracellular and extracellular power spectra of the synchronized activity showed approximately an order of magnitude more HG power than the spectra from asynchronous activity (Fig. 4A,*B*, bottom graphs; Fig. 4C,*D*; *p* < 0.05^E,F^), and the amounts of HG power in the asynchronous case trended toward baseline (Fig. 4C,*D*; Extracellular, *p* = 0.076^G^; Intracellular, *p* = 0.26^H^).

Since perfect synchrony of neuronal bursting may not be attained in real networks, we applied varying levels of jitter to the *in vitro* signals to estimate the effect of desynchronization on the preservation of HG power (Fig. 4E,*F*). As expected for signals at ∼100 Hz, both the intracellular and extracellular jittered averages showed the most dramatic decrease in power within maximum delays of 10 ms (75-80% reduction; Fig. 4E,*F*, inset).

### *In vivo* data

#### Significant amounts of HG power exist in the core

**Figure 5.**
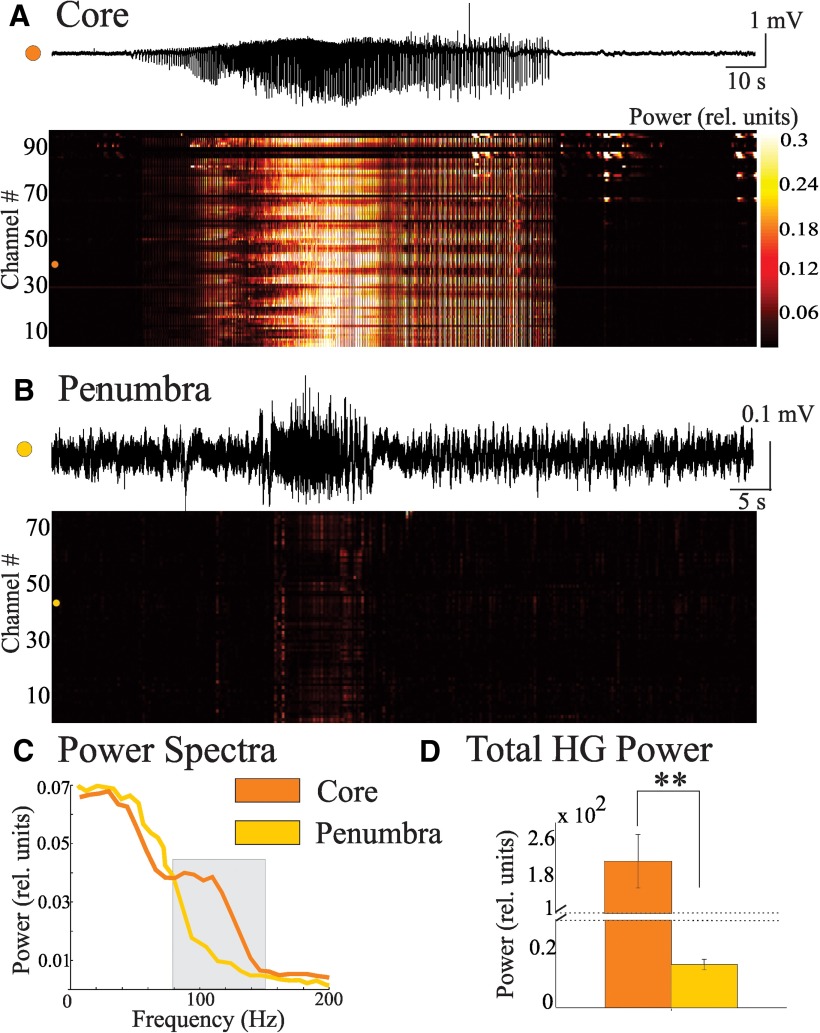
Microelectrodes in the core record higher levels of HG power compared with those in the penumbra. Seizure activity from MEA recordings was separated *post hoc* into the following two distinct territories: (1) the ictal core, defined as the cortical area where low-frequency activity is correlated with high levels of multiunit activity (two patients, four seizures); and (2) the ictal penumbra, where the low-frequency fluctuations are accompanied by uncorrelated and small changes in firing (two patients, four seizures). ***A***, Top, Example microelectrode recording from core territories during a seizure. Bottom, Heat map of HG power detected in 100 ms overlapping epochs across the seizure (50 ms overlap). Channel # corresponds to the single microelectrode channels of the MEA. ***B***, Same as in ***A*** for a recording taken from the penumbral territory. ***C***, Examples of power spectra from microelectrodes in the core (orange) and penumbra (yellow) during fully developed seizure. Note that the core shows more power in the HG band. ***D***, Bar graph of the mean and SEM of the HG power from the entire seizure for microelectrode recordings in the ictal core compared with those in the penumbra shows significantly more HG power in the core territories; note the discontinuity in the vertical axis (**p < 0.02).

The presence of HG power *in vivo* was determined by analyzing MEA recordings from patients undergoing surgical evaluation in the following two distinct territories: (1) the ictal core (two patients, four seizures); and (2) the ictal penumbra (two patients, four seizures), as previously defined by Schevon et al. (2012) and Weiss et al. (2013). Typical examples of the microelectrode activity around the seizures are shown in Figure 5, *A* and *B*. Heat maps for core and penumbral HG power are depicted in Figure 5, *A* and *B*. Figure 5C provides examples of the normalized power spectra from both territories. Across our dataset, total HG power during the seizure was significantly higher for microelectrodes in the core than in the penumbra (Fig. 5D; *p* < 0.05^I^).

#### Pseudo-ECoG signals in the core show increased synchrony and HG activity

**Figure 6.**
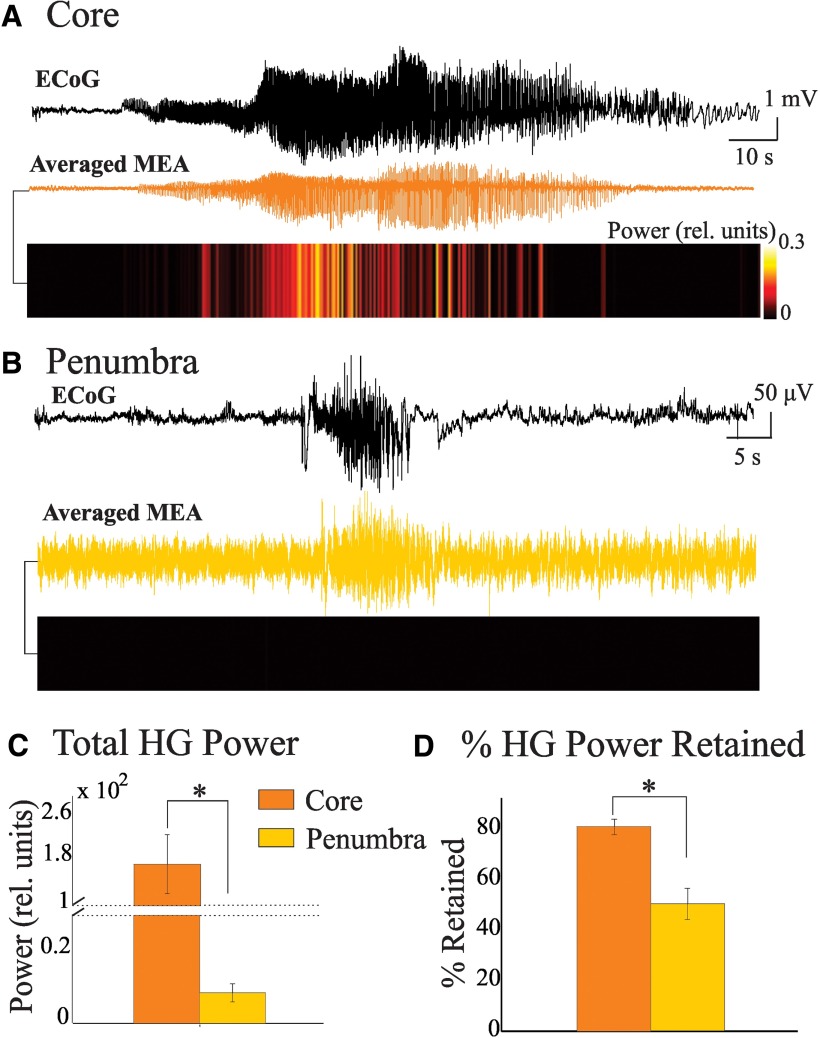
Averaged MEA activity shows HG power predominantly in the core. ***A***, Example ECoG trace recorded from a cortical grid electrode 1 cm from the MEA location (black), Pseudo-ECoG (orange) produced by averaging the microelectrode channels for a single seizure in the ictal core. Bottom, A heat map of HG power of the Pseudo-ECoG in the ictal core territory. To compute the heat map, we subdivided signals into 100 ms overlapping epochs (50 ms overlap). ***B***, Same as in ***A*** for a recording in the penumbra. ***C***, Bar graph of mean and SEM of the HG power of the Pseudo-ECoG activity across the entire seizure in core and penumbra shows more HG power in the averaged activity of the core; note the discontinuity in the vertical axis. ***D***, Since computing the averaged temporal activity takes into account differences in phase between HG activities at each microelectrode, synchrony was measured as a percentage of the HG power retained after temporal averaging compared with the averaged spectra of the individual microelectrodes (Fig. 2; Eq. 4). More power was retained in the core, suggesting a higher level of HG synchrony between these microelectrodes (*p < 0.05).

We then averaged the activity across all the microelectrodes in the 4 × 4 mm array to create a pseudo-ECoG signal (i.e. a proxy for a macroelectrode recording produced by volume conduction; Eq. 1). With the exception of the low-frequency components, our pseudo-ECoG signals were representative of the clinical ECoG recordings from the same patient, even with a distance of 1 cm between the recording sites (Fig. 6A,*B*, top traces). Evaluating the HG power within the pseudo-ECoG signal (Fig. 6A,*B*), we found that significant amounts of HG power were sustained in the core but not in the penumbral recordings (Fig. 6C; *p* < 0.05^J^). Given that our temporal averaging across the MEA takes into account phase differences between the microelectrode signals, the preservation of HG power after averaging implies that there is a larger amount of synchrony between microelectrode channels in the core than in the penumbra. We confirmed this theory by estimating the level of synchrony in both sets of data as a percentage of maximal synchrony using Equation 4 and found a significantly higher level of synchrony (mean, 78 ± 3.1%) in the core compared with the penumbra (mean, 50 ± 6.2%; Fig. 6D; *p* < 0.05^G^).

**Figure 7.**
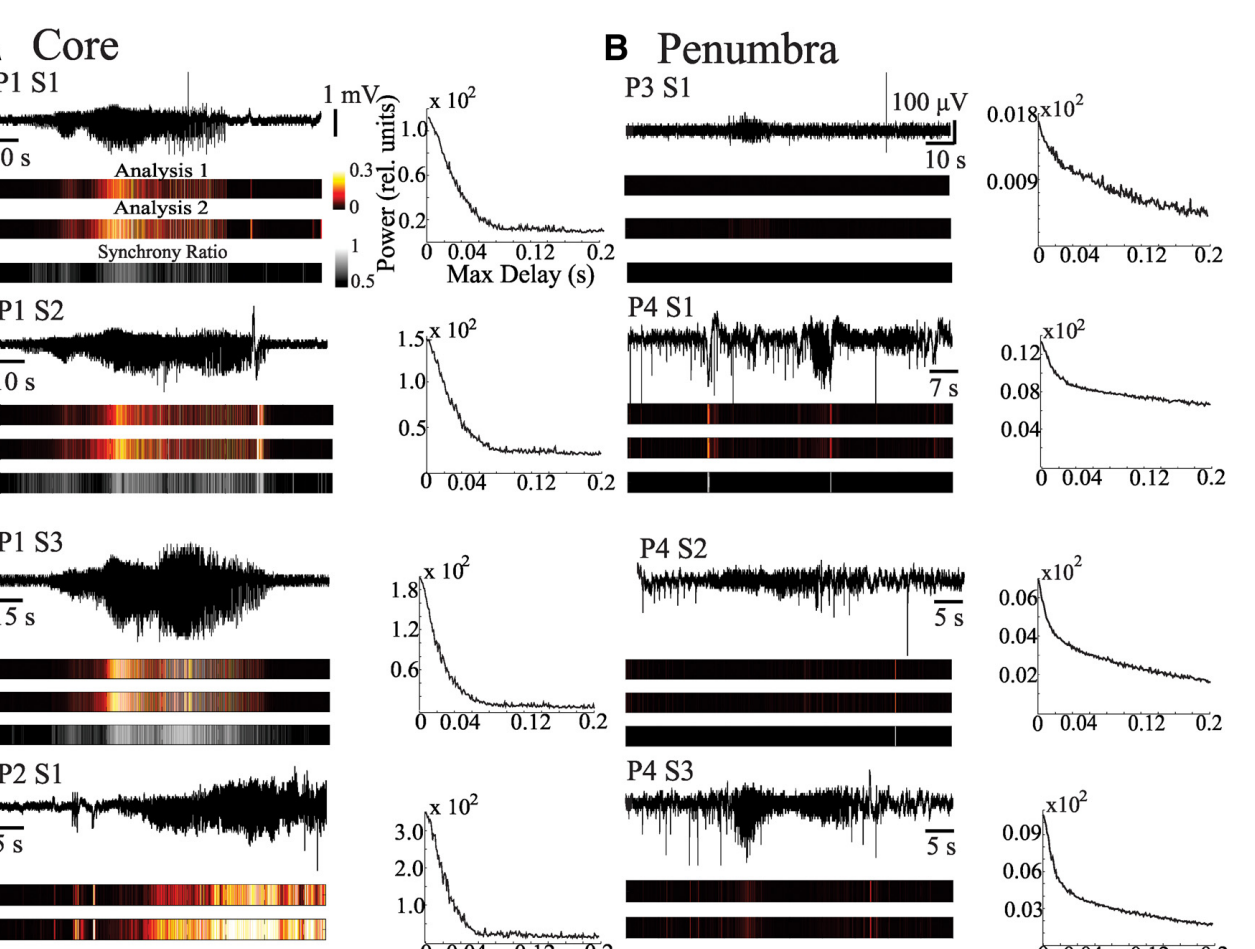
Synchrony is increased during seizure activity in the core. P, Patient number; S, seizure number. ***A***, Pseudo-ECoG recorded from ictal core territories. In the bar labeled Analysis 1, HG power is determined from the power spectrum of the time average. In contrast, in the Analysis 2 case, HG power is determined in the average spectrum of the individual power spectra of the MEA signals. The bar labeled as Synchrony Ratio represents the ratio between the two analyses (Fig. 2; Eq. 4). Heat maps and synchrony were determined in 100 ms overlapping epochs (overlap, 50 ms). The heat maps represent the averaged spectral activity, and the averaged temporal activity and their synchrony ratio. Right-hand graphs show the result of our jitter analysis, depicting the amount of HG power (ordinate) as a function of the maximum delay (abscissa). Jitter plots from core seizures show a slow decline in HG power. ***B***, Same as in ***A*** for seizures recorded from penumbral territories. Note different scales used for the ordinate across the jitter plots.

We then quantified synchrony levels with a 100 ms resolution (Fig. 7). Again, the core territories showed high levels of synchrony, as reflected by the similarities between the heat maps of the averaged spectral activity and the averaged temporal activity, and was confirmed using our synchrony ratio (Fig. 7A, color and grayscale bars). In contrast, HG activity in the penumbra showed lower levels of synchrony (Fig. 7B), reaffirming the overall result depicted in Figure 6. To explore the effects of varying levels of synchrony on the preservation of HG power at the macroscale, we applied our jitter analysis to the individual microelectrode recordings prior to creating our pseudo-ECoG (Fig. 7, graphs). The resulting jitter plots from the core showed a gradual but substantial decrease in HG power (Fig. 7A). The jittered penumbral recordings also show a slight decrease in HG power with increased desynchronization; however, energy levels were low compared with those in the core (Fig. 7B). Importantly, the effect of jitter on the decay of HG power *in vivo* (Fig. 7) clearly differs from the result obtained from the *in vitro* data (Fig. 4E,*F*).

### Computational modeling

**Figure 8.**
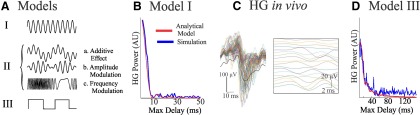
HG power observed at microelectrode and macroelectrode scales may result from different mechanisms. ***A***, Example signals of the individual generators in three types of models (I–III) we investigated in the context of the jitter analysis. Model I consists of generators of sinusoidal frequencies within the HG band. Model II represents the interaction of the HG oscillation with a sinusoidal oscillator of lower frequency. This model consisted of the following three subtypes: (1) an additive effect of the low- and high-frequency oscillations; (2) the amplitude of the HG oscillation is modulated; and (3) the HG frequency is modulated. The model III generators are nonsinusoidal, resulting in harmonics within the HG power band. ***B***, Model I, Equation 7, predicts a steep decline in the compound HG power when small amounts of jitter are added to the system. This model result closely matches the rapid depletion of HG power seen in the jitter analysis of the *in vitro* signals (Fig. 4). Both the analytical result (red) and a stochastic simulation of the same model (blue) are plotted. ***C***, Filtered microelectrode signals from *in vivo* MEA recordings at the ictal core (filter band, 80-150 Hz). This example of HG activity across microelectrodes in the MEA shows significant bursts of HG power originating from the slower dominant seizure oscillation. In addition, ongoing HG oscillations show a small amount of baseline desynchronization of ∼10 ms (inset). These delays are not unexpected for the network size involved in this measurement. Considering the dimensions of the array, 96 electrodes distributed in a 4 × 4 mm area, this corresponds to propagation rates that are bounded at ≤50 mm/s, showing a clear overlap with the range of propagation velocities between 20 and 100 mm/s observed during disinhibited slice activity (Trevelyan et al., 2006). ***D***, Prediction of model III, Equation 9. Analytic and stochastic results are plotted as in ***A***. Because of the baseline asynchrony in the *in vivo* recordings ***C***, we ignored delays of <10 ms in this model. This scenario can explain a drop-off similar to those observed in the jitter plots of the *in vivo* data (Fig. 7).

In order to investigate the differences in the slope of the *in vitro* and *in vivo* jitter plots (Figs. 4, 7, respectively), and to explain how the effects of volume conduction on locally generated signals can vary across different spatial scales, we introduce three analytical models with increasing levels of complexity (Fig. 8A). The first model is based on the average activity of a large number of identical HG oscillators. The second model includes combinations of HG and low-frequency oscillations. The third model is based on the average activity of nonsinusoidal oscillators with frequencies below the HG band. In all three models, we simulated our jitter analysis and compared the results with our experimental data.

#### Model I: pure high-gamma activity

In this model, we assume that the HG oscillations observed result from the mass activity of a large number of oscillators, where each oscillator generates a sinusoidal time series expressed as follows:

$$x\left(t+\tau_{i}\right)=\sin\left(\omega_{\gamma }\left(t+\tau_{i}\right)\right).$$

All oscillators have an identical and constant frequency, ω_γ_ (e.g., 100 Hz; Fig. 8A, Model I), where the phase differences (i.e., jitter) between the oscillators are given by ω_γ_τ*i*. We use ensemble averaging (Fig. 2) to simulate the volume conduction effects of distributed generators (governed by Eq. 1) with different degrees of synchrony (jitter). To accomplish this, we draw delays τ*i* from a uniform distribution ranging from 0 to *τ*_max_. Using this procedure, we generate an ensemble of time domain signals, *x*(*t* + τ*_i_*), that includes a sinusoidal component with a randomly applied delay. From this ensemble we can obtain an average *A*. To assess the effect of the degree of synchrony, we repeat this procedure of computing an ensemble average for a range of values for *τ*_max_ (Fig. 2A). Therefore, the average *A*(*t*, τ_max_) is both a function of time and *τ*_max_. For the stochastic simulation of this model (Fig. 8B,*D*, blue lines), we averaged an ensemble of 100 oscillators, each at the same frequency, but having randomly selected delays.

To obtain a corresponding analytical estimate of the ensemble average (Fig. 8B,*D*, red lines), we evenly sample the uniform distribution at delays τ*i*, so that 0 ≤ τ_i_ ≤ τ_max_, and τ_*i*+1_ − τ*_i_* is a constant step. If we, for simplicity of notation, set the step size to unity (i.e., express *τ*_max_ as a multiple of the step size), we obtain the following:

(5) $$A(t,\tau_{\max})=\frac{1}{\tau_{\max}}\sum_{i=0}^{\tau_{\max}}x\left(t+\tau_{i}\right),$$

with τ_max_ > 0. Because we consider a large number of generators and are interested in the frequency content resulting from their average, we may replace the summation in Equation 5 by an integral, $A(t,\tau_{\max})=\frac{1}{\tau_{\max}}\int_{0}^{\tau_{\max}}x\left(t+\tau \right)\;d\tau$. We evaluate this integral expression using $x\left(t+\tau \right)=sin(\omega_{\gamma }(t+\tau ))$, and take the Fourier transform *F*(ω) of the result. Next, we estimate the power spectrum *P*(ω) = *F*(ω)*F*(ω)* of the average and get the following:

(6) $${P\left(\omega \right)=\left(\frac{\pi }{{\omega_{\gamma }\tau }_{\max}}\right)}^{2}\left[1-e^{j\omega \tau_{\max}}\right]\;{\left[1-e^{j\omega \tau_{\max}}\right]}^{*}\;\delta \left(\omega -\omega_{\gamma }\right),$$

with δ as the Dirac impulse function and ${[1-e^{j\omega \tau_{\max}}]\;}^{*}$ denoting the complex conjugate of the expression in between the square brackets. By integrating this expression over the HG band, using Euler’s formula ($e^{j\omega \tau_{\max}}=\cos\omega \tau_{\max}+j\sin\omega \tau_{\max}$), and using the sifting property of the δ function (van Drongelen, 2007, Chapter 2), we find the expression for the power in the HG band (*P_HG_*)

(7) $${P_{HG}=4\left(\frac{\pi }{{\omega_{\gamma }\tau }_{\max}}\right)}^{2}\left[1-cos({\omega_{\gamma }\tau }_{\max})\right]$$.

We repeat computing the power in the HG band for a range of values of τ_max_ and plot *P*_HG_ as a function of the degree of synchrony, represented by the maximum delay τ_max_ (Fig. 8B). Both the analytical estimate and the simulation in the jitter plot of Figure 8B show a sharp drop in HG power with an increase in maximum delay. The expression in Equation 7 shows that the slope of the steep initial drop over which *P*_HG_ attenuates with τ_max_ depends on the frequency ω_γ_. Here we used 100 Hz as an example, but for frequencies within the HG band the steep drop occurs within a range of 6.7 ms (for 150 Hz) to 12.5 ms (for 80 Hz). We used the example of a single HG frequency since the predicted steep drop in *P*_HG_ for a signal composed of a series of oscillations within the HG band is governed by the superposition of the individual results determined by Equation 7 (i.e., the expected steep attenuation occurs in 6.7-12.5 ms, an order of magnitude of 10 ms). When compared with results from the *in vitro* dataset, this elementary model is an accurate representation of the <10 ms decline in HG power observed in our experimental jitter plots from single neurons and microscopic networks (Fig. 4E,*F*).

#### Model II: combined low-frequency and high-gamma oscillators

While Equation 7 of Model I describes the *in vitro* data accurately, it cannot explain the slower drop off observed in our jitter analysis from the *in vivo* data (Fig. 7). As HG activity has been shown to be modulated by lower frequencies in both physiological settings (Canolty et al., 2006) as well as during ictal time periods (Ibrahim et al., 2014), we modeled the case where a low-frequency sinusoidal oscillation, sin (ω*_L_t*, interacts with an oscillation in the HG band, sin (ω_γ_*t*. Within this approach, we examined the following three alternatives (Fig. 8A, Model II): (1) an additive effect between the oscillators; (2) modulation of the amplitude; and (3) frequency modulation. For each alternative, we then estimated the power in the HG band as a function of the degree of synchrony (i.e., τ_max_).

Neither the use of an additive combination nor the application of frequency modulation affects the fast drop-off in HG power described by Equation 7. In contrast, the amplitude-modulated HG signal was capable of explaining a slow drop-off as we observed in our *in vivo* data. However, this occurred only when the HG rhythms in the modeled signals were synchronized (i.e., when HG oscillators were allowed to desynchronize, a fast drop-off similar to that predicted by Equation 7 was observed.) Such a requirement of HG synchrony was found to be unrealistic since bandpass-filtered (80-150 Hz) signals demonstrated that HG activity *in vivo* still had a baseline level of asynchrony between microelectrodes (Fig. 8C). Therefore, none of these scenarios of model II can explain the slow drop-off observed in Fig. 7, and further details of this model are not reported.

#### Model III: harmonics of a nonsinusoidal signal model

Since the previous two models could not explain our *in* vivo jitter results, we then investigated the hypothesis that the relationship observed is due to harmonics that originate from nonsinusoidal oscillations. Given the transients in the filtered HG activity from the microelectrode recordings at the seizure core, it seems plausible that harmonics play a role in the generation of HG activity at the macroscopic level (Fig. 8C). This third approach was modeled with a rectangular signal *rect*(…) consisting of a series of pulses. The interval between the pulses is *T_m_*, and the duration of each pulse is *D*. Each pulse is the difference between two Heaviside functions *U*(…), as follows:

(8) $$rect\left(t\right)=\sum_{n=0}^{N}U\left(t-nT_{m}\right)-U\left(t-nT_{m}-D\right).$$

This expression represents a time series with pulses at time *nT_m_*, with *n* = 0, 1, 2, … *N*. Taking the limit of many oscillators, similar to the approach explained in Model 1, we obtained the following for the power of the harmonics in the HG band, between frequencies, *HG_l_ - HG_h_*, as a function of maximal jitter delay τ_max_, as follows:

(9) $$P_{HG}=\int_{{HG}_{l}}^{{HG}_{h}}{\left(\frac{1}{{\omega^{2}\tau }_{\max}}\right)}^{2}\sum_{n=0}^{N}{{|e}^{-j\omega (nT_{m}+\tau_{\max})}-e^{-j\omega nT_{m}}-e^{-j\omega \left(nT_{m}+D+\tau_{\max}\right)}+e^{-j\omega \left(nT_{m}+D\right)}|}^{2}\;d\omega .$$

The integral terms in Equation 9 are in the form$\int \frac{e^{-j\Delta \omega }}{\omega^{2}}d\omega =-j\Delta Ei\left(-j\Delta \omega \right)-\frac{e^{-j\Delta \omega }}{\omega }$, with Δ a delay term, and *Ei*, the exponential integral function.

Finally, in order to make the signals from our third model comparable to the *in vivo* dataset, we also took into consideration the baseline amount of asynchrony present between the *in vivo* microeletrodes (Fig. 8C, inset). Observations in our records showed a baseline jitter across the MEA of up to ∼10 ms, corresponding to a propagation velocity of <50 mm/s, which is in line with previously published propagation velocities (Trevelyan et al., 2006). Therefore, a baseline amount of jitter of 10 ms was included in our modeled signals.

The theoretical jitter results from Model III are depicted in Figure 8D and show a reasonable match for the HG–jitter relationship observed in the core (Fig. 7A). This model also appropriately describes the slow drop-off rates of the low power penumbra jitter plots (Fig. 7B), making it a suitable explanation for the effects of volume conduction on HG power at the macroscopic level.

## Discussion

We examined cellular and network epileptiform activity in human neocortex and established a relationship between paroxysmal depolarization shifts and pathological, synchronous high-gamma activity across different spatial scales. We showed that the mechanisms underlying the presence of HG activity are scale dependent. In small submillimeter networks, strict synchrony in the form of minimal phase differences between HG sinusoidal oscillators is sufficient to generate HG power in the LFP. In contrast, larger macroscopic networks can generate HG power from the harmonics of nonsinusoidal oscillators with frequencies below the HG band (40-75 Hz), and strict synchrony is not needed. These scale-dependent differences in HG activity generation emphasize that both the scale and context of the observed HG activity should be considered when applying HG power to identify critical brain regions involved in seizure generation.

We applied an established seizure model using human neocortical slices to examine and relate single-cell and network patterns during evoked epileptiform activity (Marcuccilli et al., 2010). We confirmed that neocortical HG activity can be produced in networks at a scale far below the size of the macroelectrode-through-microelectrode recordings from small slice-sized networks (Figs. 3, 4). We showed that both normally bursting (nonsaturating) and PDS-generating single neurons produced signals with similar levels of HG power (Fig. 3A,*C*). However, simultaneous LFP recordings showed that small submillimeter networks, including neurons generating PDS bursts, produced three times more HG power compared with bursting networks with nonsaturated single-cell activity (Fig. 3B,*D*,*F*). This finding suggests that the neuronal generators are more synchronized in the case of networks with PDSs, and it indicates a link between network and cellular markers of epileptiform activity, HG activity, and PDSs, respectively. While our experimental observations cannot provide direct evidence for a connection between the PDS and ictal HG activity in the epileptic core *in vivo*, the comparable amounts of HG activity in our extracellular *in vitro* recordings and microelectrode recordings provide a strong basis for our hypothesis. Furthermore, this hypothesis is supported by analogous findings in animal models (Grenier et al., 2003), and saturation effects seen during ictal activity from human MEA recordings (Meijer et al., 2015, their Figs. 1, 11).

We showed that HG preservation due to volume conduction and synchrony is associated with both experimental and clinical seizure activity. With our jitter analysis, the rapid decline in HG power with increased desynchronization of cellular generators (Fig. 4E) suggests that the level of synchrony across the PDS-generating small networks is probably high, with ≤10 ms delays. Considering the rate of neuronal conduction and transmission, synchrony levels of this order of magnitude are certainly possible within the small network recorded by a single microelectrode. However, the outcome of the jitter analysis for multiple *in vivo* microelectrode signals depicted in Figures 7 and 8 indicates that highly synchronous HG activity is clearly not a viable explanation for the presence of HG power at the macroscopic level. Obviously, at this network scale, lower levels of synchrony (above the 10 ms mark) may occur (Fig. 7). While the delays caused by volume conduction (Eq. 1) are negligible, the delays caused by neuronal conduction and synaptic transmission at this scale are not negligible and may explain the slower drop-off (Buzsáki, 2006; Nunez and Srinivasan, 2006). Therefore, it can be expected that synchrony levels within a network decrease with network size.

Using *in silico* modeling to assess differences in HG preservation across spatial scales, we were able to propose a biologically plausible explanation for the generation of HG activity in a variety of networks. Within a microscopic network, the HG sinusoidal oscillations of Model I have been shown to be capable of explaining the propagation of HG power, even with the addition of stochasticity to the system (Fig. 8B). We explored alternative models to explain the macroscopically observed HG power in the *in vivo* recordings. Model II provided an alternative theory for the macroscopic drop-off in HG power, but is not plausible upon the presence of baseline jitter *in vivo*. Thus, Models I and II fail to provide a realistic explanation for the slow drop-off in HG power. Instead, we could explain our observation of HG power drop-off in the jitter plots by harmonics originating from low-frequency nonsinusoidal fluctuations, as described by Model III (Fig. 8D). This would agree well with the previously reported presence of harmonics in seizure recordings (Foffani et al., 2007; Alvarado-Rojas et al., 2015; Fink et al., 2015).

Although we use sinusoidal oscillations in Models I and II, we do not intend to claim that the HG power in our recordings is due to pure sinusoidal signals. However, we can justify this simplification because the nonsinusoidal part of any oscillation within the HG frequency range will show up as a harmonic outside the HG band, while a slight aperiodicity or a nonsinusoidal shape of signals oscillating within the HG frequency band will still contribute to the power in that relatively wide band. Likewise, with Model III, we do not intend to suggest that the *in vivo* signals are periodic rectangular waves but aim to describe the observed drop-off of the HG power in Figure 7 by using a generic signal capable of generating harmonics. We show that adding variability to the model does not affect its prediction in a stochastic simulation of Equation 8 (Fig. 8D).

Based on previous work in the hippocampus (Bragin et al., 1999a,[Bibr B6][Bibr B7][Bibr B9]) and the timescales involved in the propagation of HG activity across spatial scales, we propose that HG generation is the result of excitatory connections in small networks with high levels of synchrony (≤10 ms of phase differences). Potential mechanisms for such a scenario include ionotropic glutamatergic transmission via the AMPA receptors (Destexhe et al., 1995; Myme et al., 2003), but also possibly direct electrical effects via gap junctions (Draguhn et al., 1998; Traub et al., 2001) or ephaptic transmission (Jefferys, 1990). Here, it must be noted that the role of gap junctions between excitatory neurons in the neocortex is not as obvious as that seen in the hippocampus (Wang et al., 2010). It has also been suggested that inhibition may play a role in HG generation (Timofeev et al., 2002; Trevelyan, 2009), but we cannot resolve effects from inhibition by GABA_A_ receptors on HG activity and PDS generation since we evoked experimental seizures by disinhibition. However, during a fully developed seizure (i.e., once ictal activity is present in the epileptic core), a principal role for inhibitory mechanisms is unlikely, since there is a significant failure of inhibition that is thought to be the very cause of seizure propagation and activity in this area (Schevon et al., 2012; Weiss et al., 2013; Meijer et al., 2015).

Although HG activity can be generated by a variety of physiological and pathological mechanisms, our data show that epileptiform activity is strongly associated with HG activity. The HG power in both microelectrode and macroelectrode measurements allows for the identification of the ictal core, while the low-frequency signals, such as those currently used for localization, may represent both core and penumbra territories. HG power is therefore a sensitive and specific biomarker for the identification of core seizure territories, but the ictal context in which it is observed must be considered.

## Synthesis

The decision was a result of the Reviewing Editor Muriel Thoby-Brisson and the peer reviewers coming together and discussing their recommendations until a consensus was reached. A fact-based synthesis statement explaining their decision and outlining what is needed to prepare a revision is listed below. The following reviewers agreed to reveal their identity: William Stacey, David Mogul.
